# The Story So Far: How Embodied Cognition Advances Our Understanding of Meaning-Making

**DOI:** 10.3389/fpsyg.2017.01315

**Published:** 2017-07-31

**Authors:** Cedric Galetzka

**Affiliations:** Division of Cognitive Sciences, University of Potsdam Potsdam, Germany

**Keywords:** embodied cognition, abstract concepts, language, mental simulation, action words

## Abstract

Meaning-making in the brain has become one of the most intensely discussed topics in cognitive science. Traditional theories on cognition that emphasize abstract symbol manipulations often face a dead end: The symbol grounding problem. The embodiment idea tries to overcome this barrier by assuming that the mind is grounded in sensorimotor experiences. A recent surge in behavioral and brain-imaging studies has therefore focused on the role of the motor cortex in language processing. Concrete, action-related words have received convincing evidence to rely on sensorimotor activation. Abstract concepts, however, still pose a distinct challenge for embodied theories on cognition. Fully embodied abstraction mechanisms were formulated but sensorimotor activation alone seems unlikely to close the explanatory gap. In this respect, the idea of integration areas, such as convergence zones or the ‘hub and spoke’ model, do not only appear like the most promising candidates to account for the discrepancies between concrete and abstract concepts but could also help to unite the field of cognitive science again. The current review identifies milestones in cognitive science research and recent achievements that highlight fundamental challenges, key questions and directions for future research.

## Introduction

Where do words get their meaning from? This so-called grounding problem is at the core of the current theoretical debate within the cognitive sciences. Cognitivism, on the one hand, assumes that the human mind works like a computer. Accordingly, meaning is supposed to arise from abstract symbol manipulations in mentalese, the hypothesized language of thought. However, it is unclear how symbols in mentalese can ever come to mean anything (Harnad, 1990; Bergen, 2012). Famously illustrated in the Chinese room thought experiment, Searle (1980) argued that computers relying on symbol manipulations alone would never be capable of reaching semantic understanding. Embodied approaches, on the other hand, emphasize that abstract symbols necessarily need to be grounded in our experiences with the world. That is, information from the senses should be central to the way we think and understand. And mental simulation, the reactivation of sensorimotor information, is thought to be the crucial mechanism (Barsalou, 2008). But just how far does the explanatory umbrella of embodied cognitive science actually reach? The current article aims to identify key research areas in modern cognitive science that highlight the importance of the embodiment idea but also its limits.

## Sensorimotor Components of Concept Knowledge

Due to its crucial role in meaning-making, language can be considered a prime research area for the advancement of embodied theories on cognition. Embodied approaches predict that semantic understanding results from re-experiencing what words refer to. In other words, sensorimotor areas involved in perception and action should overlap with brain regions that are active during language comprehension (Barsalou, 2008; Bergen, 2012). Indeed, in an fMRI study, Hauk et al. (2004) demonstrated that seeing action verbs, such as ‘kick’ or ‘lick,’ elicited similar brain activation patterns in the motor and premotor areas compared to the actual movements the words referred to. The authors argued that semantic processing relies on a Hebbian association learning mechanism whereby words get linked to the perceptions and actions they correlate with. Conversely, TMS stimulation of the somatosensory cortex related to the arms or legs facilitated recognition of action verbs involving movement of the respective extremities (Pulvermüller et al., 2005). Pulvermüller (2005) argued that these findings are inconsistent with a cognitivist view of cognition in which meaning is processed entirely in a single hub, most often hypothesized to be the anterior temporal lobes (ATL; for a recent review, see Lambon Ralph et al., 2016). Instead, semantic understanding seems to be partly related to a distinct motor component.

Further, language comprehension involves the reconstruction of the context from previous perceptual experiences (Bergen, 2012). For example, Zwaan et al. (2002) presented subjects with sentences implying varying shapes of relevant objects (eagle in the sky vs. eagle in the nest) and observed congruency effects in response to pictures that matched the implied orientation of the object. That is, participants responded faster to pictures depicting eagles with outspread wings when they had read sentences about eagles in the sky. Similarly, Pecher et al. (2003) showed that participants were slower to verify tactile properties for a given word pair (peanut butter – sticky) when the previous trial required visual property verification (highway sign – green) compared to another tactile property verification. The authors argued that these modality switch costs in language comprehension are akin to costs in perceptual processing. Indeed, in a follow-up fMRI experiment, the same word pairs elicited activation in brain areas corresponding to the respective perceptual modalities (Simmons et al., 2003). Recently, Scerrati et al. (2016) further demonstrated that switching the mode of presentation (visually vs. aurally) also leads to a processing cost for congruent word pairs. The researchers concluded that if a purely perceptual manipulation is able to interfere with conceptual processing, sensorimotor activation must be involved in language comprehension. This underscores the idea that semantic processing is tightly linked to perceptual systems.

## Necessity or Simply a By-Product?

Are these activations of the sensorimotor system merely associative in nature or constitutive of meaning-making? According to embodied theories of cognition, representations are modal in that they are grounded in sensorimotor areas of the brain. As such, activation of sensorimotor systems should occur early in the processing stream. Indeed, Hauk et al. (2008) observed motor areas to become active as soon as 200–300 ms after stimulus presentation. However, Mahon (2015) maintains that previous results simply reflect an initial activation of amodal representations spreading down to sensorimotor systems. Relevant to that claim, Papeo et al. (2014) used rTMS to investigate action verb processing in the left posterior middle temporal gyrus (lpMTG), a region previously shown to be involved in conceptual processing but distinct from sensorimotor areas. The researchers observed syntactic deficits for action verbs over nouns when rTMS was applied to the lpMTG. More importantly, perturbation to the lpMTG reduced activation in the primary motor cortex. Papeo et al. (2014) argued that meaning processing is hierarchically organized with amodal representations mediating activation in motor areas. Herein lies the first challenge for embodied theories of cognition, namely to make explicit the exact time course and mechanism of spreading activation from sensory-motor to language areas of the brain (Fischer and Zwaan, 2008).

In contrast to Mahon’s (2015) epiphenomenalism claim, the sensorimotor system does seem to represent a necessary component for language comprehension (Jirak et al., 2010). Neininger and Pulvermüller (2003) studied the effects of word processing in a lexical decision task in patients with lesions either to their frontal or the temporo-occipital brain areas. Recognition accuracy for action-related verbs was impaired for patients with damage to frontal cortical regions, commonly associated with the planning, execution and control of motor responses. Interestingly, nouns related to visual features were less accurately recognized in temporo-occipital patients. These data suggest a double dissociation in syntactic understanding between action-related verbs and nouns associated with perceptual qualities. The former seems to rely on activation of the motor system whereas the latter partly rests on sensory areas of the brain (Neininger and Pulvermüller, 2003; Pulvermüller and Fadiga, 2010). However, more recent results cast doubt on whether the motor system is crucially involved in language comprehension. Kemmerer et al. (2013) compared performance on a semantic similarity judgments task in Parkinson’s disease patients on and off dopaminergic treatment. Importantly, while patients were slower to distinguish between action and non-action words, their accuracy was similar to the control subjects and unaffected by medication. The authors argued against a strong reading of the embodied cognition hypothesis, according to which the intactness of the motor system is the sole factor in language processing.

Does damage to sensorimotor systems result in substantial conceptual knowledge loss? According to Binder and Desai (2011), selective sensorimotor perturbations merely lead to mild semantic processing deficits. Instead, damage to the temporal lobes has been observed to be more strongly associated with far-reaching semantic impairments, such as semantic dementia (Jefferies and Lambon Ralph, 2006). Moreover, Mahon and Caramazza (2005) reported apraxia patients whose object recognition abilities were spared despite a lack of appropriate motor response production. This distinction is supposed to highlight that information about possible motor interactions cannot be crucially involved in concept representations (Mahon, 2015). Similarly, Vannuscorps and Caramazza (2016) recently showed that reasoning about upper limb movements is not restricted in patients with upper limb dysplasia despite apparent deficits in mental simulation via sensorimotor activation. In contrast, Hauk and Tschentscher (2013) argue for a more fine-grained distinction according to which impairments in sensorimotor functioning are regularly accompanied by concrete syntactic knowledge impairments. Abstract conceptual information, however, does not seem to rely on these sensorimotor areas to the same extent. The second challenge then, is to examine to which degree sensorimotor areas are necessary for semantic knowledge activation (cf. Fischer and Zwaan, 2008). In order to account for these inconsistencies, embodied cognition approaches need to specify how knowledge is abstracted from sensorimotor input.

Recent progress on these key questions has been somewhat inconclusive. Dalla Volta et al. (2014) compared the spatiotemporal dynamics of concrete vs. abstract word processing using high density EEG. The researchers found indication of early sensorimotor activation but only for concrete words. Similarly, Innocenti et al. (2014) found that TMS impulses applied over the motor cortex only interfered with action-related words (see also Repetto et al., 2013). The authors argued that this effect might also be susceptible to familiarity since there was no indication for the involvement of the motor cortex during a second round of the experiment. However, Dreyer et al. (2015) compared the performance of two patients with varying lesions to the motor cortex and found evidence for sensorimotor grounding. When brain damage was limited to somatotopic hand areas, words associated with tools were most affected. Damage to the supplementary motor cortex resulted in impairments in recognizing abstract emotional words. Moreover, Vukovic et al. (2017) used rTMS during a semantic decision task. When applied over the motor cortex, participants had difficulties identifying action-related words but processing abstract words was facilitated. These results largely bolster the proposed causal role of the sensorimotor regions in concrete word processing but leave abstract words still in need of further clarification.

Metaphors provide a means to examine how abstract concepts are grounded in sensorimotor systems. Lakoff and Johnson (1999) argued that metaphor usage mediates conceptual understanding via sensorimotor activation reflecting literal word meaning. For instance, the expression “to grasp an idea” would allow comprehension of the abstract concept of understanding via somatotopic activation of hand motor areas. In fact, Boulenger et al. (2009) compared the somatotopic activity of the motor cortex during a lexical decision task in which subjects were presented with sentences depicting literal and idiomatic verb usage (e.g., ‘Pablo kicked the ball’ vs. ‘Pablo kicked the habit’). The fMRI results supported an embodied approach to language comprehension in that both, literal and idiomatic meanings elicited activation of the respective motor cortical area. However, in another study by Aziz-Zadeh et al. (2006), metaphors based on motor movements did not elicit any comparable brain activation patterns. Aziz-Zadeh and Damasio (2008) reasoned that highly familiar metaphors might not draw on the same motor resources anymore when compared to newly established metaphors. This conclusion corresponds closely to an account of conflation learning in which abstract concepts are initially mingled together with sensorimotor experiences in an early stage of development. For example, children often experience affection as being accompanied by physical warmth. These associations then persist up through adulthood and continue to shape abstract concepts in more differentiated ways (Johnson, 1999; Lakoff and Johnson, 1999, see also Casasanto, 2017). In fact, Desai et al. (2013) found that sensorimotor activation in response to action words is heavily context dependent. In their fMRI study, the researchers observed a gradual decline of involvement of sensorimotor areas during the presentation of literal, metaphoric, idiomatic and abstract action word meanings.

## The Need for Integration Areas

Criticism on the metaphor approach often focuses on the limited explanatory scope for high-level cognitive processes. Abstract concepts that do not seem to share corresponding sensorimotor components, such as numbers, democracy or truth, constitute a major challenge for embodied cognitive science (Dove, 2016). In order to capture the full complexity of abstract concepts, researchers hypothesized that semantic memory is dependent on activation from integration areas in the brain. In fact, Damasio (1989) originally proposed the existence of so-called convergence zones. These are supposed to be located proximal to sensorimotor areas and are thought to comprise highly interconnected neuronal assemblies arranged in hierarchically organized semantic levels (Damasio and Damasio, 1994). Moreover, Thompson-Schill (2003) hypothesized that anterior shifts of neural activity within these convergence zones are associated with increasing semantic complexity. For example, Gainotti et al. (2013) argued that abstract knowledge about different word categories might be represented by a different combination of sensory modalities. Biological concepts might rely more on information from the visual and other perceptual modalities whereas visual and motor knowledge is more important for artifacts. Hence, more abstract categorization of biological and artifact concepts might emerge at the intersection of the ventral or dorsal stream and the sensorimotor cortex, respectively. Yang et al. (2017) measured neuronal spike activity in sensorimotor cortex and adjacent parietal regions during an action verb reading task. The researchers observed a gradual shift from neurons predominantly exhibiting verb meaning mapping in sensorimotor cortex to abstract verb category processing by neurons in parietal regions. The authors argued for a distributed neural network in close correspondence to weak embodiment approaches. However, abstract processing of verb category did not depend on prior activation of sensorimotor areas, thereby pointing to parallel processing of abstract and concrete semantics. Another future challenge for embodied cognitive science is then not only to identify convergence zones close to sensorimotor areas but also the extent to which these zones operate independently of sensorimotor input (Mahon and Caramazza, 2008; Meteyard et al., 2012).

Another prominent theory of semantic memory assumes that meaning arises from converging activity in a single hub, the ATL. In their framework of controlled semantic cognition, Lambon Ralph et al. (2016) suggest that the ATL alone integrates multimodal input to form abstract and generalizable concepts instead of multiple convergence zones. Their reasoning is partly based on studies with patients who developed semantic dementia following damage to the ATL, which results in marked deficits in semantic memory for a wide range of concepts (Lambon Ralph and Patterson, 2008). In a recent fMRI study, Coutanche and Thompson-Schill (2014) investigated the link between the processing of different object features and the ATL. The researchers found that the ATL stored a higher-level representation of lower-level object features, such as color and shape, that was activated during an object retrieval phase. Moreover, Jang et al. (2017) used multielectrode arrays during a word-pair association task and observed increased firing rates in the middle temporal gyrus, a subregion of the ATL, when subjects were asked to recall the specific associations. The researchers interpreted this finding as evidence for the neural coding of higher-order representations of concept associations within the ATL. However, as Binder and Desai (2011) note, the framework of controlled semantic cognition fails to explain the mechanisms behind multimodal integration in brain areas such as the lateral and ventral temporal lobe and the inferior parietal lobe. Future research could focus on the hierarchy in semantic memory and possible interactions between the ATL and other convergence zones.

## Conclusion

To sum up, research on language comprehension can provide deep insights about the fundamental aspects of knowledge acquisition. Sensorimotor systems have a well-documented impact on the processing of concrete action-related words (Hauk et al., 2004; Pulvermüller et al., 2005). As a result, disembodied theories seem unlikely to be able to offer a full account of semantic processing (Meteyard et al., 2012) Abstract concepts, however, have divided the field of embodied cognition. Strong embodiment approaches fail to provide a comprehensive account of abstraction mechanisms. Metaphors alone seem too limited to explain the complexity of abstract concepts and related brain activity distinct from sensorimotor regions (Dove, 2016). Weak embodiment approaches emphasizing integration areas as the basis for abstract concepts seem to be the safest bet at the moment (Meteyard et al., 2012). Such a middle path also seems likely to reconcile previous amodal and embodied theories on cognition. (Barsalou, 2016). Future research will need to address neural mechanisms for meaning-making and the relation between convergence zones, the ATL and sensorimotor areas. Key questions will concern how sensorimotor brain areas become directly involved in the formation of abstract concepts or simply function as a gateway to higher cognition. Ultimately, the embodiment idea represents a key step in cognitive science and will continue to shape our understanding of how the brain achieves meaning.

## Author Contribution

The author confirms being the sole contributor of this work and approved it for publication.

## Conflict of Interest Statement

The author declares that the research was conducted in the absence of any commercial or financial relationships that could be construed as a potential conflict of interest.
