# Corrigendum: The Story So Far: How Embodied Cognition Advances Our Understanding of Meaning-Making

**DOI:** 10.3389/fpsyg.2017.01813

**Published:** 2017-10-13

**Authors:** Cedric Galetzka

**Affiliations:** Division of Cognitive Sciences, University of Potsdam, Potsdam, Germany

**Keywords:** embodied cognition, abstract concepts, language, mental simulation, action words

In the original article, I neglected to include the funder **(Deutsche Forschungsgemeinschaft)** and **(Open Access Publishing Fund of the University of Potsdam)** to CG. The author apologizes for this error. This does not change the scientific conclusions of the article in any way.

## Conflict of interest statement

The author declares that the research was conducted in the absence of any commercial or financial relationships that could be construed as a potential conflict of interest.

